# The effect of high-dose vitamin D supplementation on hepcidin-25 and erythropoiesis in patients with chronic kidney disease

**DOI:** 10.1186/s12882-022-03014-z

**Published:** 2023-01-25

**Authors:** Kristin Danielson Pistis, Per-Anton Westerberg, Abdul Rashid Qureshi, Soheir Beshara, Gunnar Sterner, Peter Bárány, Torbjörn Linde

**Affiliations:** 1 Renal Medicine, CLINTEC, Karolinska Institutet, Stockholm, Sweden; 2 Medical Sciences, Uppsala University, Uppsala, Sweden; 3 Department of Medicine, Åland’s Central Hospital, 22100 Mariehamn, Finland; 4 Molecular Medicine and Surgery, Karolinska Institutet, Stockholm, Sweden; 5 Renal Medicine, Skåne University Hospital, Malmö, Sweden

**Keywords:** Chronic kidney disease, Anemia, Vitamin D, Iron, Hepcidin

## Abstract

**Background:**

Hepcidin is considered to play a central role in the pathophysiology of renal anemia. Recent studies in healthy individuals have demonstrated a suppressive effect of vitamin D (VD) on the expression of hepcidin. In this post-hoc analysis based on a randomized controlled study, we evaluated the effect of supplementing chronic kidney disease (CKD) patients (stage G3-G4) with a high daily dose of native VD on serum levels of hepcidin-25, the hepcidin/ferritin ratio, as well as on markers of erythropoiesis.

**Methods:**

Patients with CKD stage G3-G4 included in a double blind, randomized, placebo (PBO) controlled study with available hepcidin measurements were analyzed. Study subjects received either 8000 international units (IU) of cholecalciferol daily or PBO for 12 weeks. We evaluated the change in markers of hepcidin expression, erythropoiesis, and iron status from baseline to week 12 and compared the change between the groups.

**Results:**

Eighty five patients completed the study. Calcitriol, but not 25-hydroxyvitamin D (25(OH) D), was inversely correlated with serum levels of hepcidin-25 (rho = -0,38; *p* =  < 0, 01 and rho = -0,02; *p* = 0, 89, respectively) at baseline. Supplementation with VD significantly raised the serum concentration of serum 25(OH)D in the treatment group (from 54 (39–71) to 156 (120–190) nmol/L; *p* =  < 0, 01)) but had no effect on any of the markers of hepcidin, erythropoiesis, or iron status in the entire cohort. However, we did observe an increase in hemoglobin (HB) levels and transferrin saturation (TSAT) as compared to the PBO group in a subgroup of patients with low baseline 25(OH)D levels (< 56 nmol/L). In contrast, in patients with high baseline 25(OH)D values (≥ 56 nmol/L), VD supplementation associated with a decrease in HB levels and TSAT (*p* = 0,056) within the VD group in addition to a decrease in hepcidin levels as compared to the PBO group.

**Conclusion:**

High-dose VD supplementation had no discernible effect on markers of hepcidin or erythropoiesis in the entire study cohort. However, in patients with low baseline 25(OH)D levels, high-dose VD supplementation associated with beneficial effects on erythropoiesis and iron availability. In contrast, in patients with elevated baseline 25(OH)D levels, high-dose VD supplementation resulted in a decrease in hepcidin levels, most likely due to a deterioration in iron status.

## Introduction

Anemia is a frequent finding in chronic kidney disease (CKD) and it is associated with increased mortality and morbidity rates [1, 2]. In addition to a relative deficiency of erythropoietin (EPO), iron deficiency (ID), characterized by either *absolute* or *functional* ID, constitutes a common contributing factor to renal anemia [3]. Whereas absolute ID is characterized by depleted iron reserves, functional ID denotes replete iron stores but insufficient iron mobilization to meet the need for efficient erythropoiesis [3].

Hepcidin, the central regulator of iron homeostasis, is considered to play a key role in the development of functional ID in CKD [3]. It regulates plasma iron availability by its interaction with ferroportin, an iron transport protein localized on the surface of iron-exporting cells. By binding to ferroportin, iron release from iron exporting cells is disabled [4]. The net result is a redistribution of iron from plasma to iron stores which contributes to the development of iron restricted erythropoiesis. In addition to its role in limiting iron availability of the bloodstream, hepcidin has been shown in in vitro experiments to hamper the proliferation and survival of erythroid progenitor cells in an environment of low erythropoietin concentration, possibly constituting a second mechanism of action relevant to the development of renal anemia [5].

The bioactive isoform of hepcidin is hepcidin-25 (HEP-25). Positive regulators of hepcidin expression comprise tissue iron levels, iron availability, and inflammatory activity whereas erythropoietic activity and hypoxia decrease its synthesis [4, 6].

Hepcidin is to large extent filtered freely by the glomeruli and clearance occurs by degradation in the proximal tubuli in addition to cellular endocytosis at its site of action [7, 8]. Consequently, in CKD, several studies (although not all) report hepcidin levels to be increased as compared to healthy individuals [9–11]. Chronic inflammation may be an additional reason for the observed elevated levels of hepcidin in patients with CKD [7, 12].

However, in patients with *absolute* ID, hepcidin levels remain reflective of the size of iron reserves (i.e. remain low) independent of the glomerular filtration rate (GFR) and degree of inflammation [9, 13, 14] and therefore hepcidin also assumes characteristics of a diagnostic marker of ID [15–17].

The strong influence of iron reserves on hepcidin expression, shown in healthy individuals as well as in patients with CKD [11, 13, 18–20], demonstrates the importance of relating levels of HEP-25 to the size of iron reserves and also consider the hepcidin/ferritin ratio (H/F ratio), which reflects the adequacy of hepcidin expression in relation to iron stores [21].

Based on its described mode of action, animal experiments demonstrating improvements of renal anemia with hepcidin ablation, in addition to supportive epidemiological data, hepcidin has gained interest as a potentially modifiable target for alleviating renal anemia [19, 22, 23].

A recently identified suppressor of hepcidin expression is vitamin D (VD), which through its downstream metabolites 25-hydroxyvitamin D (25(OH)D) and 1,25-dihydroxyvitamin D (1,25(OH)_2_D), was shown in vitro to exert a direct inhibitory effect on hepcidin mRNA expression in cultured hepatocytes [24]. Further support of this notion has been lended by in vivo experiments in which healthy individuals showed a decrease in serum levels of HEP-25 24 h after ingestion of a large bolus dose of 100, 000 IU of ergocalciferol [24]. Moreover, observational studies have also found low metabolites of VD (25(OH)D and/or 1, 25 (OH)_2_D) to independently and negatively associate with serum levels of HEP-25 in CKD, and further, with the occurrence of anemia in patient cohorts from various clinical contexts [25–28].

Only a limited number of interventional studies evaluating the suppressive effect of nutritional VD on serum levels of HEP-25 in non-dialysis CKD patients have been undertaken. Apart from a trial in pediatric CKD patients (stage G2-G5) that was unable to demonstrate a suppressive effect on serum levels of HEP-25 after 12 weeks of VD supplementation, studies in the adult CKD population evaluating nutritional VD as a potential modifier of serum HEP-25 in a placebo (PBO) controlled manner is scarce [29]. In this post-hoc analysis we therefore aimed to investigate the potential of nutritional VD to modify levels of HEP-25 and the H/F ratio in adult patients with CKD G3–G4. We hypothesized that treatment with a high daily dose of cholecalciferol would result in a decrease in serum levels of HEP-25 and the H/F as compared to the PBO group and thereby translate into favorable changes in erythropoietic activity.

## Materials and methods

### Study subjects and study design

This was originally a double-blind, randomized, PBO controlled study (EudraCT 2011–002,586-38, date of registration: 2011–08-24) with the primary aim of assessing the efficacy of a high daily dose of cholecalciferol on the development of secondary hyperparathyroidism. The results from this study have been published previously [30]. The present post-hoc analysis was based on data collected from those study subjects who fulfilled the criteria for inclusion, were randomized to treatment, and for whom measurements of HEP-25 were available at baseline and at week 12 [30].

In brief, patients with CKD G3—G4 and secondary hyperparathyroidism were recruited and randomized to either treatment with cholecalciferol, 8,000 IU per day (Vitamin D3 Forte Renapharma) for 12 weeks, or to PBO.

Patients were eligible for enrollment in the original study if they had a serum level of parathyroid hormone (PTH) above 6.8 pmol/L, 25(OH)D below 75 nmol/L, an estimated glomerular filtration rate (eGFR) quantified with the Modification of Diet in Renal Disease formula between the level of 15 and 59 mL/min/1.73 m^2^, and were between 18–85 years of age. Exclusion criteria comprised an assumed need for kidney replacement therapy (KRT) during the following 6 months, ionized calcium exceeding 1.30 mmol/L, carrier of a kidney transplant, pregnancy, acute or chronic systemic inflammatory disease at enrollment, disease with major affect on mineral metabolism, primary hyperparathyroidism, malabsorption, or active malignancy. Additional exclusion criteria comprised treatment with calcimimetics, paricalcitol, or supplementation with ergocalciferol or cholecalciferol in a dose higher than 400 IU/day. A low and stable dose of oral 1-alfacalcidiol was accepted given that the dose was unchanged during the study.

The study was compliant with the Declaration of Helsinki. The Ethical Review board in Uppsala (Ref 2011/313) as well as the Swedish Medical Products Agency approved the study protocol.

### Laboratory protocol

Blood was collected after an overnight fast and routine biochemical analyses were carried out locally by standard laboratory methods. Quantification of serum 25(OH)D for screening purposes was performed locally with high-performance liquid chromatography mass spectrometry (HPLC–MS) in Malmoe and Gothenburg while a chemiluscent method (Diasorin) was used in Uppsala. Baseline and week-12 measurements of 25(OH)D were all analyzed centrally by HPLC–MS. Measurements of serum HEP-25 were carried out with mass spectrometry that specifically measures HEP-25. This method has a lower limit of quantification of 0,5 nmol/L, an intra-assay coefficient of variation of 6%, and an inter-assay coefficient of variation of 8%. Finally, the quantification of intact fibroblast growth factor 23 (FGF-23) was performed with an enzyme-like immunosorbent method (Kainos, Japan).

In healthy individuals it has been shown that serum ferritin explains 60% of the variance in serum levels of hepcidin-25 [20]. It is therefore relevant to consider not only serum levels of HEP-25 but also the H/F ratio in order to relate the level of HEP-25 to the size of the iron reserves as the ratio better reflects the adequacy of hepcidin expression in relation to body iron reserves [21], (www.hepcidinanalysis.com).

### Data collection

Data were collected on demography, cause of CKD, concomitant medical treatment of relevance, and eGFR.

### Statistical analyses

Normally distributed data are shown as means ± standard deviation (SD), whereas skewed distributions are presented as medians with interquartile ranges. Categorical data are presented as percentages. Differences between groups at baseline were assessed using a Student’s T-test for comparing means, a Mann–Whitney-U test for skewed data or a Chi-square test for categorical variables. Associations between parameters at baseline were analyzed with a Spearman’s rank correlation test. A multiple linear regression model was applied to assess the individual impact of various baseline parameters on serum hepcidin-25 concentrations and the H/F ratios. To test if there was a difference within the groups between baseline and week 12 a Wilcoxon rank sum test was utilized. For differences between groups in change from baseline to week 12, a Mann–Whitney-U test was applied. A *p*-value < 0.05 was considered statistically significant. To investigate whether the severity of VD insufficiency would influence the results, we stratified the patients based on the median level of S-25(OH)D at baseline of the patients who completed the study. The statistical analyses were carried out using SPSS version 27.

## Results

### Baseline characteristics

At baseline, measurements of HEP-25 were available for 88 study subjects (43 from the VD group and 45 from the PBO group). The corresponding numbers at week 12 were 42 study subjects from the treatment group and 43 study subjects from the PBO group. Baseline characteristics for the cohort comprising patients with available measurements of HEP-25 are presented in Table 1. The two groups were similar regarding demographic, clinical, and biochemical data aside from two exceptions; the proportion of male sex was higher in the PBO group (78% versus 56%; *p* = 0,03) and the H/F ratio was higher in the treatment group as compared to the PBO group; 43 (23–60) versus 31 (17–44); *p* = 0,02.

**Table 1:** Characteristics of patients included in the hepcidin cohort^a^

Patient characteristics	Cholecalciferol (*n* = 43)	PBO (*n* = 45)	*p*-value
Male sex, n (%)	24 (56)	35 (78)	**0,03**
Age (years)	62 (± 16)	64 (± 14)	0,59
BMI (kg/m^2^)	28,0 (± 4,8)	28,8 (± 7,2)	0,53
eGFR (ml/min/1,73m^2^)	32 (± 13)	30 (± 12)	0,47
CKD stage G3, n (%)	19 (44)	16 (36)	0,47
CKD stage G4, n (%)	24 (56)	29 (64)	-
*Cause of CKD*			
Diabetes mellitus, n (%)	13 (30)	13 (29)	0,89
Glomerulonephritis, n (%)	8 (19)	8 (18)	0,92
PKD, n (%)	5 (12)	1 (2)	0,08
Nephrosclerosis, n (%)	11 (26)	12 (27)	0,91
Other, n (%)	6 (14)	11 (24)	0,21
Oral iron, n (%)	9 (21)	4 (9)	0,13
Intravenous iron, n (%)	1 (2)	0 (0)	-
ESA, n (%)	4 (9)	5 (9)	0,8
Hemoglobin (g/L)	129 (± 16)	132 (± 16)	0,49
Hematocrit (%)	39 (± 5)	40 (± 5)	0,37
Erythrocytes (10^12^/L)	4,2 (± 0,53)	4,4 (0,59)	0,33
Ferritin (µg/L)	121 (76–256)	123 (60–237)	0,78
TSAT (%)	27 (± 9)	26 (± 10)	0,60
S-iron (µmol/L)	16 (± 5)	16 (± 5)	0,9
Hepcidin (nmol/L)^b^	5,2 (2,8–10)	4,4 (1,4 – 7,3)	0,18
Hepcidin/ferritin ratio^c^	43 (23–60)	31 (17–44)	**0,02**
CRP (mg/L)	2,0 (0,71 – 5,5)	2,4 (1,3 – 5,6)	0,34
IL-6 (ng/L)	1,8 (1,0 – 3,7)	3,0 (1,2 – 4,5)	0,26
25(OH)D (nmol/L)	56 (± 22)	58 (± 22)	0,74
1,25(OH)_2_ (pmol/L)	58 (26–90)	53 (34–90)	0,97
Phosphate (mmol/L)	1,2 (± 0,28)	1,2 (± 0,28)	0,46
Calcium (mmol/L)	2,2 (± 0,14)	2,3 (± 0,11)	0,18
FGF-23 (pg/ml)	92 (71 – 163)	130 (68–179)	0,40

*BMI* Body mass index, *eGFR* Estimated glomerular filtration rate, *PKD* Polycystic Kidney Disease

ESA, erythropoiesis-stimulating agents; TSAT, transferrin saturation; CRP, high-sensitivity C-reactive protein; IL-6, interleukin-6; 25(OH)D, 25-hydroxyvitamin D; 1,25(OH)_2,_ 1,25-dihydroxyvitamin D; FGF-23: fibroblast growth factor 23

^a^Values are presented as median (interquartile range), mean (± SD), or absolute numbers (%). *p*-values denotes differences between the treatment group and the placebo group. *p*-values below 0,05 are shown in bold print

^b^Reference values for serum hepcidin-25 (nmol/L; median, 95% reference range) for the general population with mass spectrometry (WCX-TOF MS): men 4.7 (< 0.5–15.5); women 3.8 (< 0.5– 15.4). Reference: www.hepcidinanalysis.com

^c^Reference values for hepcidin-25/ferritin ratio (pmol/µg; median, 95% reference range) for the general population with mass spectrometry (WCX-TOF MS): men, 28,2 (3,1–92,7); pre-menopausal women, 37,6 (3,2– 176,4); post-menopausal women, 42,7 (9,6–150,9). Reference: www.hepcidinanalysis.com

### Univariate and multivariate associations with baseline hepcidin concentrations

At baseline, S-HEP-25 correlated positively with S-ferritin, transferrin saturation (TSAT) and negatively with S-calcitriol, eGFR, and HB (Table 2). The H/F ratio was only associated with S-ferritin.

**Table 2:** Univariate Spearman rank correlations with hepcidin and the hepcidin/ferritin ratio at baseline

	**Age**	**eGFR**	**IL-6**	**ferritin**	**CRP**	**25(OH)D**	**phosphate**	**calcitriol**	**Hb**	**TSAT**	**FGF-23**	**calcium**	**S-iron**
Hepcidin	-0,1	**-0,21**	0,04	**0,67**	0,11	-0,02	0,11	**-0,38**	**-0,25**	**0,21**	0,20	-0,13	-0,02
H/F ratio	-0,13	-0,04	-0,14	**-0,22**	0,07	0,01	0,12	-0,20	-0,16	-0,07	-0,06	-0,03	-0,12

Numbers in bold denotes p < 0,05. *e GFR* estimated glomerular filtration rate, *TSAT* Transferrin saturation, *HB* Hemoglobin, *H/F ratio* hepcidin/ferritin ratio, *CRP* High-sensitivity C-reactive protein, *IL-6* Interleukin-6, *25(OH)D* 25-hydroxyvitamin D, *1,25(OH)*_*2*,_ 1,25-dihydroxyvitamin D, *FGF-23* fibroblast growth factor 23

In a multiple linear regression model (Table 3) showing the impact of various parameters on S-HEP-25, S-ferritin, TSAT, and high-sensitivity C-reactive protein (CRP) was shown to positively influence S-HEP-25 whereas a trend for a negative association with HEP-25 was seen for S-calcitriol and HB (*p* = 0,06 for both).

**Table 3:** Multiple linear regression model with the 1-SD of hepcidin increase as dependent variable at baseline, adjusted R^2^ = 0, 43

per 1-SD of Hepcidin	Beta (*p*-value)
per 1-SD increase of age	-0,13 (0,16)
gender, male vs female	-0,07 (0,48)
per 1-SD increase of CRP	0,30 (**0,00**)
per 1-SD increase of S-ferritin	0,48 (**0,00**)
per 1-SD increase of S-calcitriol	-0,19 (0,06)
per 1-SD increase of HB	-0,20 (0,06)
per 1-SD increase of e GFR	0,03 (0,78)

*CRP* high-sensitivity C-reactive protein, *HB* Hemoglobin, *e GFR* estimated glomerular filtration rate. Numbers in bold denotes p < 0, 05. Substituting S-ferritin for TSAT showed TSAT to associate with S-hepcidin (β = 0, 31; *p* = 0, 00; R^2^ = 0, 30)

### Change in laboratory markers between baseline and week 12

As shown in Table 4, no change between baseline and week 12 was observed for S-HEP-25, the H/F ratio, HB levels, S-ferritin, TSAT, CRP or IL-6 in the treatment group. As reported previously, S-25(OH)D increased significantly in the treatment group (from 54 nmol/L (39, 71) to 156 nmol/L (120, 190); *p* < 0,001). S-1,25(OH)_2_ increased from 56 (26, 92) to 86 (60, 138) pmol/L in the treatment group (*p* = 0,001). Increases were also observed over time for CRP, S-phosphate, and FGF-23 in the VD group of which the latter also increased significantly in the PBO group.

**Table 4:** Median ± interquartile range for various laboratory variables before and after 12 weeks of treatment with cholecalciferol or PBO for patients with measurements of hepcidin-25 available at baseline and week 12 (*n* = 85)

**Parameter**	Cholecalciferol (*n* = 42)			PBO (*n* = 43)		
	**Baseline**	**Week 12**	***p*-value**	**Baseline**	**Week 12**	***p*-value**
**Hemoglobin (g/L)**	126 (119, 137)	130 (115, 138)	0,45	135 (120, 145)	131 (118, 140)	0,09
**Hematocrit (%)**	38 (36, 41)	37 (35, 41)	0,90	41 (37, 43)	40 (36, 42)	0,06
**Erythrocytes (10**^**12**^**/L)**	4,1 (3,9, 4,6)	4,0 (3,8, 4,5)	0,25	4,3 (4,0, 4,8)	4,3 (3,9, 4,8)	0,08
**25(OH)D (nmol/L)**	54 (39, 71)	156 (120, 190)	** < 0,01**	59 (39, 72)	57 (42, 79)	0,12
**1,25(OH)**_**2**_**D (pmol/L)**	56 (26, 92)	86 (60, 138)	** < 0,01**	53 (34, 90)	59 (38, 82)	0,95
**hepcidin-25 (nmol/L)**	5,3 (3,0, 10,5)	4,8 (3,0, 9,8)	0,28	4,5 (1,9, 7,5)	3,7 (2,2, 6,6)	0,66
**hepcidin / ferritin ratio**	46 (23, 61)	45 (24, 63)	0,20	31 (16, 42)	33 (19, 51)	0,40
**ferritin (µg/L)**	124 (77, 257)	140 (73, 246)	0,46	142 (73, 239)	115 (67, 255)	0,28
**CRP (mg/L)**	2,0 (0,71, 4,4)	4,0 (1,3, 7,6)	**0,03**	2,2 (1,3, 5,4)	3,4 (1,2, 7,7)	0,06
**TSAT (%)**	26 (19, 34)	26 (19, 32)	0,24	26 (19, 32)	23 (18, 31)	0,07
**S-iron (µmol/L)**	17 (13, 21)	14 (11, 19)	0,14	17 (13, 19)	15 (11, 19)	**0,047**
**IL-6, (ng/L)**	1,8 (1,0, 3,5)	2,1 (1,1, 4,2)	0,10	3,0 (1,2, 4,5)	2,8 (1,5, 4,7)	0,15
**eGFR (mL/min/1,73m**^**2**^**)**	29 (22, 40)	30 (21, 42)	0,15	28 (20, 40)	28 (17, 38)	**0,02**
**Calcium (mmol/L)**	2,25 (2,17, 2,32)	2,23 (2,16, 2,29)	0,93	2,27 (2,21, 2,35)	2,23 (2,17, 2,31)	** < 0,01**
**Phosphate (mmol/L)**	1,2 (1,0, 1,3)	1,3 (1,1, 1,4)	**0,04**	1,11 (1,0, 1,3)	1,3 (1,0, 1,4)	0,11
**FGF-23 (pg/mL)**	92 (68, 164)	122 (70, 194)	** < 0,01**	131 (70, 181)	108 (81, 216)	**0,22**
***Patients with 25-(OH)D* < *56 nmol/L***	**Cholecalciferol (*n* = 22)**			**PBO (*n* = 19)**		***p*-value**
**Hemoglobin (g/L)**	122 (117, 135)	126 (113,138)	0,22	138 (117, 150)	131 (114, 144)	0,07
**Hematocrit (%)**	36 (35, 41)	37 (35, 41)	0,07	41 (36, 44)	40 (35, 44)	**0,02**
**Erythrocytes (10**^**12**^**/L)**	4,1 (3,6, 4,6)	4,1 (3,8, 4,6)	0,44	4,4 (4,1, 5,1)	4,3 (3,8, 4,9)	**0,02**
**25(OH)D (nmol/L)**	39 (31–50)	124 (106, 158)	** < 0,01**	37 (31, 43)	42 (38, 51)	**0,02**
**1,25(OH)**_**2**_**D (pmol/L)**	39 (9–77)	81 (48, 122)	** < 0,01**	49 (35, 76)	60 (44, 86)	0,65
**hepcidin-25 (nmol/L)**	6,2 (2,8, 12)	6,1 (3,2, 14)	0,78	4,5 (1,9, 7,1)	3,0 (2,2, 5,5)	0,72
**hepcidin / ferritin ratio**	47 (27, 55)	45 (29, 67)	0,88	32 (18, 54)	32 (22, 51)	0,98
**ferritin (µg/L)**	138 (80, 341)	149 (83, 328)	0,75	133 (76, 238)	113 (79, 186)	0,27
**CRP (mg/L)**	1,9 (0,8, 4,4)	4,4 (1,6, 7,7)	**0,02**	3,3 (2,0, 10,1)	5,5 (1,5, 21,5)	0,18
**TSAT (%)**	24 (19, 34)	25 (19, 32)	0,67	25 (16, 32)	21 (14, 25)	**0,01**
**S-iron (µmol/L)**	16 (12, 17)	15 (11, 20)	0,74	17 (12, 19)	15 (10, 15)	**0,02**
**IL-6 (ng/L)**	2,8 (0,9, 4,3)	3,4 (1,6, 5,5)	0,06	3,3 (1,6, 4,5)	3,7 (1,9, 8,4)	0,07
**e GFR (mL/min/1,73m**^**2**^**)**	29 (22–39)	31 (20–44)	0,17	34 (28, 47)	34 (21, 41)	**0,03**
**Calcium (mmol/L)**	2,20 (2,03–2,27)	2,23 (2,13–2,30)	0,18	2,27 (2,20, 2,30)	2,24 (2,15, 2,28)	**0,01**
**Phosphate (mmol/L)**	1,1 (1,0, 1,3)	1,3 (1,1, 1,5)	**0,03**	1,2 (1,0, 1,4)	1,3 (1,0, 1,5)	0,11
**FGF-23 (pg/mL)**	91 (70, 78)	127 (70, 275)	**0,02**	112 (53, 164)	101 (64, 166)	0,5
***Patients with 25-(OH)D* ≥ *56 nmol/L***	**Cholecalciferol (*n* = 20)**			**PBO (*n* = 24)**		
**Hemoglobin (g/L)**	129 (121, 141)	126 (116, 140)	**0,01**	131 (119, 140)	132 (119, 137)	0,59
**Hematocrit (%)**	39 (36, 42)	37 (35, 42)	**0,049**	38 (37, 42)	39 (36, 42)	0,64
**Erythrocytes (10**^**12**^**/L)**	4,3 (3,9, 4,6)	4,0 (3,8, 4,4)	**0,02**	4,2 (3,9, 4,7)	4,2 (3,9, 4,7)	0,97
**25(OH)D (nmol/L)**	71 (61, 81)	184 (156, 220)	** < 0,01**	72 (64, 87)	76 (59, 86)	0,49
**1,25(OH)**_**2**_**D (pmol/L)**	67 (45, 103)	102 (72, 152)	** < 0,01**	60 (33, 95)	54 (36, 81)	0,72
**hepcidin-25 (nmol/L)**	5,1 (3,1, 7,2)	4,3 (2,4, 7,0)	**0,04**	4,3 (1,6, 8,4)	4,3 (2,1, 7,1)	0,37
**hepcidin / ferritin ratio**	40 (21, 81)	44 (18, 54)	**0,04**	29 (15, 41)	35 (18, 52)	0,29
**ferritin (µg/L)**	108 (76, 242)	109 (72, 209)	0,56	142 (41, 276)	137 (59, 272)	0,65
**CRP (mg/L)**	2,4 (0,7, 7,1)	3,4 (0,9, 7,4)	0,55	1,7 (0,9, 4,4)	2,6 (0,8, 5,9)	0,16
**TSAT (%)**	28 (20, 34)	22 (17, 26)	0,056	27 (19, 35)	26 (20, 38)	0,88
**S-iron (µmol/L)**	17 (13, 22)	13 (12, 17)	0,1	17 (13, 20)	16 (13, 21)	0,67
**IL-6 (ng/L)**	1,4 (1,0, 2,5)	1,7 (1,0, 2,5)	0,57	2,0 (0,95, 4,3)	2,3 (1,2, 4,3)	0,82
**e GFR (mL/min/1,73m**^**2**^**)**	30 (24, 44)	30 (25, 42)	0,68	21 (18, 32)	24 (16, 35)	0,24
**Calcium (mmol/L)**	2,27 (2,22, 2,37)	2,22 (2,19, 2,29)	0,052	2,27 (2,21, 2,39)	2,23 (2,17, 2,31)	0,01
**Phosphate (mmol/L)**	1,2 (1,0, 1,3)	1,3 (1,1, 1,3)	0,68	1,1 (0,9, 1,3)	1,1 (1,0, 1,3)	0,46
**FGF-23 (pg/mL)**	100 (59, 157)	106 (69, 175)	**0,01**	139 (78, 252)	145 (84, 258)	0,35

Values are presented as median and interquartile range. *p*-values refer to changes over time from baseline to week 12 within each group. *p*-values in bold denotes a value < 0,05. Sub-group analyses are based on patients with baseline values of 25-hydroxyvitamin D below, and above (or equal to), the median value of 56 nmol /L, respectively. e GFR, estimated glomerular filtration rate; TSAT, transferrin saturation; CRP, high-sensitivity C-reactive protein; IL-6, interleukin-6; 25(OH)D, 25-hydroxyvitamin D; 1,25(OH)_2,_ 1,25-dihydroxyvitamin D; FGF-23: fibroblast growth factor 23

### Difference in change between the cholecalciferol group and the placebo group

As shown in Table 5, there was no significant difference between the groups in change over time from baseline to week 12 in either marker of hepcidin. The change in HB, S-ferritin, TSAT, CRP and IL-6 did not differ between groups. A greater change was observed for S-25(OH)D in the treatment group as compared to the PBO group; 93 (67, 143) versus 2 (-9, 17) nmol/L, *p* < 0,01. Likewise, the change in 1,25(OH)_2_ was greater in the treatment group as compared to the PBO group; 34 (15, 61) versus -1 (-15, 13) pmol/L, *p* < 0,01.

**Table 5:** Difference in change between the treatment group and the PBO group for different laboratory variables (*n* = 85)

Difference between baseline and week 12	Cholecalciferol (*n *= 42)	PBO (*n* = 43)	*p*-value
**Hemoglobin (g/L)**	0 (-5,—2)	-2 (-8, 3)	0,37
**Hematocrit (%)**	0,0 (-1,1, 1,0)	-1 (-2, 1)	0,26
**Erythrocytes (10**^**12**^**/L)**	0,0 (-0,2, 0,1)	0,0 (-0,2, 0,1)	0,69
**25(OH)D, nmol/L**	93 (67, 143)	2 (-9, 17)	** < 0,01**
**1,25(OH)**_**2**_**D (pmol/L)**	34 (15, 61)	-1 (-15, 13)	** < 0,01**
**Hepcidin (nmol/L)**	-0,3 (-2,7, 1,0)	0,4 (-1,9, 1,8)	0,22
**Hepcidin/ferritin ratio**	-6 (-23, 6)	3 (-14, 16)	0,13
**Ferritin (µg/L)**	-5 (-30, 16)	-4 (-31, 12)	0,73
**TSAT (%)**	-1 (-7, 4)	-1 (-8, 3)	0,77
**S-iron**	-2 (-4, 2)	-1 (-4, 2)	0,82
**CRP (mg/L)**	0,6 (-0,2, 1,9)	0,4 (-0,7, 2,6)	0,90
**IL-6 (ng/L)**	0,2 (-0,25, 1,0)	0,3 (-0,4, 1,4)	0,77
**e GFR (mL/min/1,73m**^**2**^**)**	-1 (-4, 2)	-1 (-4, 0)	0,97
**Calcium (mmol/L)**	-0,01 (-0,06, 0,06)	-0,04 (-0,10, 0,01)	**0,03**
**Phosphate (mmol/L)**	0,08 (-0,10, 0,18)	0,08 (-0,09, 0,16)	0,66
**FGF-23 (pg/mL)**	21 (-6, 39)	10 (-21, 33)	0,23
***Patients with 25(OH)D* < *56 nmol/L***	Cholecalciferol (*n* = 22)	PBO (*n* = 19)	
**Hemoglobin (g/L)**	2 (-2, 6)	-2,0 (-14, 1)	**0,03**
**Hematocrit (%)**	1 (-0,3, 2)	-1,0 (-4, 0)	**0,01**
**Erythrocytes (10**^**12**^**/L)**	0,0 (-0,1, 0,2)	-0,1 (-0,4, 0,0)	**0,03**
**25(OH)D, nmol/L**	84 (66, 115)	6 (-1, 13)	** < 0,01**
**1,25(OH)**_**2**_**D (pmol/L)**	34 (10, 59)	0 (-8, 14)	** < 0,01**
**Hepcidin (nmol/L)**	0,0 (-2,8, 2,6)	0,3 (-3,2, 1,4)	0,89
**Hepcidin/ferritin ratio**	0 (-12, 15)	0 (-7, 20)	0,92
**Ferritin (µg/L)**	-8 (-31, 29)	-11 (-56, 20)	0,55
**TSAT (%)**	6 (-3, 6)	-4 (-11, 0)	**0,047**
**S-iron (µmol/L)**	-1 (-3, 3)	-2 (-7, 2)	0,11
**CRP (mg/L)**	0,8 (-2,4, 3,6)	0,6 (-0,7, 8)	0,93
**IL-6 (ng/L)**	0,5 (-0,1, 1,3)	0,9 (-0,3, 4,2)	0,49
**e GFR (mL/min/1,73m**^**2**^**)**	-2 (-7, 1)	-2 (-5, 0)	0,60
**Calcium (mmol/L)**	0,02 (-0,05, 0,15)	-0,04 (-0,10, 0,23)	0,02
**Phosphate (mmol/L)**	0,1 (0,0, 0,3)	0,1 (-0,1, 0,2)	0,53
**FGF-23 (pg/mL)**	25 (-11, 66)	18 (-37, 37)	0,40
***Patients with 25(OH)D* ≥ *56 nmol/L***	Cholecalciferol (*n* = 20)	PBO (*n* = 24)	
**Hemoglobin (g/L)**	-3 (-6, 1)	-1 (-6, 3)	0,24
**Hematocrit (%)**	-1,0 (-2,0, 0)	0 (-2, 1)	0,20
**Erythrocytes (10**^**12**^**/L)**	-0,10 (-0,2, 0,0)	0,0 (-0,20, 0,16)	0,09
**25(OH)D, nmol/L**	113 (83, 151)	-5 (-12, 17)	** < 0,01**
**1,25(OH)**_**2**_**D (pmol/L)**	33 (16, 64)	-1 (-16, 13)	** < 0,01**
**Hepcidin (nmol/L)**	-0,8 (-2,9, 0,2)	0,4 (-1,3, 2,2)	**0,04**
**Hepcidin/ferr ratio**	-10 (-27, 3)	6 (-13, 10)	**0,02**
**Ferritin (µg/L)**	-5 (-30, 13)	0 (-25, 10)	0,9
**TSAT (%)**	-3 (-8, 1)	0 (-5, 3)	0,1
**S-iron (µmol/L)**	-2 (-5, 1)	0,0 (-3, 2)	0,22
**CRP (mg/L)**	-0,1 (-0,3, 1,1)	0,4 (-0,5, 1,2)	0,71
**IL-6 (ng/L)**	0,2 (-0,6, 0,9)	0,2 (-0,5, 1,2)	0,92
**e GFR (mL/min/1,73m**^**2**^**)**	-1 (-3, 2)	-1 (-2, 1)	0,73
**Calcium (mmol/L)**	-0,03 (-0,06, 0,02)	-0,05 (-0,11, 0,01)	0,59
**Phosphate (mmol/L)**	0,1 (-0,1, 0,1)	0,1 (-0,1, 0,2)	0,88
**FGF-23 (pg/mL)**	21 (-2, 27)	9 (-18, 31)	0,31

Values are presented as median and interquartile range. *p*- values refer to differences in change over time between the treatment group and the PBO group. *p*- values in bold print denotes a *p*-value < 0,05. Sub-group analyses are based on patients with baseline values of 25-hydroxyvitamin D below and above (or equal to) the median value of 56 nmol/L, respectively. e GFR, estimated glomerular filtration rate; TSAT, transferrin saturation; CRP, high-sensitivity C-reactive protein; IL-6, interleukin-6; 25(OH)D, 25-hydroxyvitamin D; 1,25(OH)_2_, 1,25-dihydroxyvitamin D; FGF-23: fibroblast growth factor 23

### Sub-group analyses

To investigate whether the severity of vitamin D insufficiency would influence the results, we performed subgroup analyses in patients with S-25(OH)D levels below or above (and equal to), respectively, the median level of baseline S-25(OH)D (56 nmol/L) of the 85 patients that completed the study (depicted in Table 4 and 5). In the low 25(OH)D group, the HB, hematocrit (HCT), erythrocyte count (RBC), and TSAT, increased significantly as compared to the PBO group. No change in the hepcidin markers was observed. In the PBO group, all markers of erythropoiesis decreased over time (the HB-value by a trend; *p* = 0,07) as did TSAT. FGF-23 and CRP increased in the treatment group but remained unchanged in the PBO group.

In the subgroup of high 25(OH)D levels, all markers of erythropoiesis decreased significantly over time within the treatment group, and TSAT decreased by a trend (*p* = 0,056), whereas FGF-23 increased in the VD group. These changes were absent in the PBO group. In the same subgroup, serum HEP-25 and the H/F ratio decreased over time, and exhibited a greater decrease as compared to the PBO group. Of note, S-ferritin remained unchanged in both subgroups.

We also assessed whether patients on the higher range of S-HEP-25 (> 4,8 nmol/L) levels would exhibit a greater decrease in hepcidin markers as compared to the PBO group. However, none of the markers of hepcidin, erythropoiesis, or iron availability, changed more over time as compared to the PBO group (data not shown) even though the H/F ratio decreased over time within the VD group (from 47 (32–73) to 45 (25–56); *p* = 0,01) as did S-iron levels and TSAT alike (from 17 (12–19) to 13 (10–16) µg/L for S-iron; *p* = 0,01, and from 27 (19–34) to 22 (17–26) % for TSAT; *p* = 0,02 respectively). A parallel decrease over time was observed for the RBC (from 4,1 (3,9–4,6) to 4,0 (3,7–4,4) 10^12^/L; *p* = 0,037).

## Discussion

In the present post-hoc analysis study based on CKD 3 – 4 patients with vitamin D insufficiency, we assessed the effect of a high daily dose of native vitamin D_3_ on serum levels of HEP-25 and the H/F ratio. 12 weeks of treatment with VD did neither result in a change in S-HEP-25, nor in the H/F ratio, or in any other marker reflecting erythropoiesis or iron availability. However, in a subgroup comprising patients with 25(OH)D levels on the lower range (< 56 nmol/L), the HB levels, the HCT, and the RBC, in addition to the TSAT, increased as compared to the PBO group. In contrast, in patients with high baseline 25(OH)D levels, all markers of erythropoiesis (HB, HCT, and RBC) decreased over time, in parallel to a decrease in hepcidin levels as compared to the PBO group; the latter most likely as a reflection of a deterioration in iron availability.

We based our hypothesis on in vitro data demonstrating that treatment of cultured hepatocytes with the VD metabolites 25(OH)D and 1,25(OH)_2_D decreased the expression of HEP-25, in addition to in vivo experiments in non-anemic, healthy individuals showing a decrease in serum levels of HEP-25 after ingestion of a large bolus dose of VD [24, 31]. Based on these reports, we hypothesized that treatment of CKD patients with a high daily dose of native VD could modify serum levels of HEP-25 and potentially alleviate iron-restricted erythropoiesis commonly observed in CKD [4].

In the present study we found no effect on markers of hepcidin when a cut-off level of< 75 nmol/L was applied and this agrees with a previous study in pediatric non-dialysis CKD patients assessing the effect of native VD on circulating levels of HEP-25 [29].

Our results deviate from the ones reported from studies carried out in healthy individuals and there are some potential reasons as to why the outcome of VD-mediated hepcidin suppression may differ between healthy individuals and CKD-patients.

Firstly, in the present analysis, we studied patients with CKD who frequently have elevated levels of HEP-25 as compared to healthy individuals [9, 10, 13]. Secondly, different from healthy individuals, patients with CKD often present with some degree of chronic inflammation which may contribute to increased serum levels of HEP-25 [12, 32]. However, S-HEP-25 values of the present cohort (Table 1) lied within the normal reference range for HEP-25 (men 0.5–15.5; women < 0.5– 15.4) and this may have impeded a VD suppressive effect on HEP-25 levels although we did observe a decrease over time in the H/F ratio (but not in S-HEP-25) in the subgroup of patients on the higher range of S-HEP-25 levels (above the median; 4,8 nmol/L) of the VD group. This decrease was however paralleled by a decrease in S-iron and TSAT and may therefore have been secondary to a drop in iron availability. Nevertheless, the absence of an effect on HEP-25 in patients with moderate CKD and normal S-HEP-25 values does not exclude an effect in patients with more severe CKD in whom HEP-25 levels tend to be higher than in earlier stage CKD patients [14].

In the experiments in healthy individuals, the evaluation of HEP-25 levels was performed shortly after ingestion of the VD bolus dose; 24 h and 1 week respectively, as opposed to after 12 weeks in our study [24, 31]. Due to potential adaptiations in the hepcidin-ferroportin axis gradually induced by VD supplementation, the timing of the evaluation may play a role as to why no difference was observed in our study after 12 weeks. An acute decrease in HEP-25 levels is expected to result in a prompt increase in plasma iron availability, in itself a potent stimuli for elevated hepcidin expression, leading hepcidin levels to recover [33]. This notion is supported by a recent study comprising critically ill patients supplemented with 100.000 IU of cholecalciferol for 5 consequetive days [34]. HEP-25 was decreased compared to the PBO group when assessed one week from baseline. At week two and three, however, differences between the groups were no longer present even though a positive effect on HB levels remained.

The original aim of the present study was to assess the suppressive effect of VD on parathyroid hormone levels and the 25(OH)D threshold for inclusion was in line with previous KDOQI guidelines recommending a target value of 75 nmol/L in S-25(OH)D, based on the rational of minimizing the risk of secondary hyperparathyroidism [35]. Current versions of the guidelines recommend using the same strategy that would be applied for the general population, without stating a cut-off, whereas the European Renal Best practice group advocates VD supplementation when 25(OH)D levels lie below 31 nmol/L, but however states that optimal management of 25(OH)D levels between 31 – 75 nmol/L is unclear. There is thus inconsistency regarding optimal 25(OH)D levels in CKD, but also for the general population [36]. The Endocrine Society defines VD insufficiency at 25(OH)D levels below 75 nmol/L, whereas the Institute of Medicine (IOM), on the other hand, advocates 25(OH)D levels > 50 nmol/L [37, 38].

Further, it has been questioned whether a *single,* universal threshold of serum 25(OH)D exists above which VD supplementation has beneficial health effects, and hypothesized that disease-specific cut-offs may be more appropriate [39]. This notion is based on the assumption that the conversion of 25(OH)D to 1,25(OH)_2_D is regulated differently depending on the tissue in question and that the minimum serum 25(OH)D level required to meet the demand of a specific tissue therefore may differ depending on the tissue of study [35, 39–41].

In light of the inconsistency in regard to how VD insufficiency should be defined, and potential inconsistencies due to differences in tissue requirements, we stratified the patients based on the median baseline S-25(OH)D level (< 56 or ≥ 56 nmol/L) and found a greater increase in HB, HCT, RBC, and TSAT in the low 25(OH)D group as compared to the PBO group whereas S-HEP-25 and the H/F ratio remained unchanged (Table 5).

Subgroup analyses must be interpreted restrictively. However, a beneficial effect of VD on erythropoietic activity could potentially be attributed to the stimulatory effect of calcitriol on the proliferation of erythroid precursors, which may be mediated through the stimulation of erythropoietin receptor expression [42, 43]. This is in line with a number of small, non-PBO controlled studies that have demonstrated treatment with *active* analogues of VD to associate with improvements in HB levels and ESA resistance in CKD [44–46].

The mechanism by which iron availability improved in the low 25(OH)D group is not clear but may have involved a VD-induced suppression of hepcidin expression although no longer discernable at week 12 due to adaptive changes in the hepcidin/ferroportin axis. Erythroferrone, a hormone expressed by erythroblast during increased erythropoietic activity with the down-stream effect of accelerating iron absorption and iron recycling by limiting hepcidin expression, may also have played a role in improving iron status given the parallel improvement in erythropoietic markers [47]. Interestingly, the potential benefit of VD stimulation on iron status was limited to plasma iron availability, rather than stored iron, given that S-ferritin remained unchanged.

Most, but not all, previous studies assessing the effect of *native* vitamin D for the alleviation of anemia in non-renal clinical settings were however unable to show improvements in anemia related parameters, consequent with the results of a meta-analysis on the same topic [48]. However, of note, the majority of these studies applied either a threshold of 75 nmol /L for inclusion (as in the present study) or did not restrict the study population to individuals with vitamin D insufficiency, which may account for these null-findings. However, two studies based on VD-insufficient patients with hypertension, and heart failure, respectively, reporting no effect on HB levels after VD supplementation, was not able to find an effect even in subgroups of patients with severe VD deficiency (< 25 and < 30 nmol/L, respectively) [49, 50]. The authors hypothesized that high baseline HB levels (144 g/L) may have limited further HB improvements in one of the studies, and that baseline calcitriol levels (81, 6 pmol/L) were above the threshold over which a further increase would stimulate erythropoiesis in the second study. Comparability with trials carried out in non-CKD patients assessing the stimulatory effect of VD on erythropoiesis are complicated by the fact that the increase in calcitriol levels upon stimulation with native VD tends to be steeper in CKD patients (in whom the activity of renal 1,25-hydroxylase is substrate-dependent to a greater extent than in healthy individuals) as compared to patients with non-CKD in whom calcitriol levels plateau at a lower level of 25(OH)D [41, 51, 52]. This may be of importance given that the correlation between calcitriol and HB levels is seemingly stronger as compared to that between 25(OH) D levels and HB levels [26, 27, 53].

Whereas favourable effects on erythropoiesis and iron availability were observed in the low 25(OH)D group, an opposing effect was discerned in the high 25(OH)D group in the sense that all markers of erythropoiesis (HB, HCT and RBC) and TSAT (*p* = 0,056) decreased over time within the VD group of patients with baseline 25(OH)D levels ≥ 56 nmol/L. However, no difference was observed as compared to the PBO group in patients with 25(OH) levels above 56 nmol/L and its relation to treatment can therefore not be ascertained but nevertheless constitutes an unexpected adverse finding.

Moreover, in the high baseline 25(OH)D group, serum HEP-25 and the H/F ratio decreased as compared to the PBO group. These changes are likely not the result of an inhibitory effect of VD metabolites on the HAMP gene but rather a reflection of a decrease in iron availability observed within the VD group, possibly contributing to the changes in markers of erythropoiesis in addition to a potential increased susceptibility of erythrocytes to eryptosis due to excessive VD ingestion [54]. The underlying mechanism mediating a potential association between VD supplementation and deterioration in iron status is not entirely clear but may be related to elevations in FGF-23 observed in the treatment group. Animal studies support inverse associations between FGF-23 levels and iron availability and erythropoietic activity [55, 56] and clinical, prospective trials have coupled total FGF-23 levels to the risk of incident anemia [57, 58].

Based on the aforementioned findings, it may be questioned whether VD may have bidirectional effects on iron status, a phenomenon observed within other clinical contexts in which VD supplementation has been assessed as well. For instance, some meta-analyses suggest VD in combination with calcium (but not VD alone) to reduce the risk of hip-fractures [59–61] whereas some recent studies indicate that the intake of large VD doses may associate with an increased risk of falls and fractures [62–66], an undesirable effect that women may be at particular risk for [63]. A negative impact of VD on muscle performance, amongst others, have been suggested as explanation for these unexpected results [67, 68]. Moreover, prolonged intake of large VD doses has further been associated with bone density losses in women, in particular in younger women [69]. Whether these aforementioned adverse effects on musculoskeletal endpoints constitute a down-stream effect of a VD-induced deterioration in iron status and consequent deacceleration of erythropoiesis, or rather results from other non-erythroid VD signaling pathways, remains to be elucidated in future studies. Regardless, anemia has been linked to the risk of falling in elderly, and iron deficiency and anemia has been associated with the presence of osteoporosis [70–72]. Further, animal experiments and human cohort studies suggest ID to have detrimental effects on muscle health known to impact the risk of falls [73–75]. At any rate, adverse effects on iron status may be more harmful to women than to men, given that women have lower iron reserves than men, even after menopause, which could explain the particular vulnerability of high dose VD regimens in females [21]. In CKD-patients, poor iron availability not only increases the risk of anemia and anemia- related complications but also augments the risk of an unfavourable outcome [76, 77].

We are not aware of any studies reporting a deterioration in iron status although a couple of case reports have described anemia in association to VD intoxication [78, 79]. However, the decrease in serum HEP-25 and H/F ratio in the high 25(OH)D group paralleled by a deterioration in markers of erythropoies, and TSAT, over time may reflect a similar phenomenon observed in a recent randomized, PBO-controlled study in which 96 ESA-treated hemodialysis (HD) patients were supplemented with either native VD_3_ or PBO showing lower hepcidin levels, and a higher degree of ESA resistance, as compared to the PBO group at 6 months [80]. There was no difference in absolute HB values or traditional iron markers however. The fact that all patients were treated with ESA, a potent suppressor of hepcidin, complicates comparisons with the present study in which the majority of patients was ESA-free [13].

At any rate, it may be that CKD patients are more vulnerable to adverse effects of high-dose VD supplementation than non-CKD patients given that calcitriol levels tend to increase more sharply upon stimulation with native VD than in individuals without CKD [51, 52].

In regard to variables potentially reflecting a pro-inflammatory effect, S-phosphate, CRP, and S-FGF-23 increased within the treatment group, but not as compared to the PBO group, in which FGF23 also increased, raising questions regarding causality. Nevertheless, S-phosphate and S-FGF-23 are molecules that risk accumulation upon VD supplementation and both have potential direct, or indirect, pro-inflammatory effects which may be reflected by the increase over time in CRP, although a trend for increase in CRP (*p* = 0,06) was observed in the PBO group too [81, 82]. FGF-23 elevations constitute a potentially undesirable effect of VD supplementation for which CKD patients may be at particular risk [83].

On a final note, baseline levels of HEP-25 levels associated inversely with S-calcitriol and HB (by a trend for significance; *p* = 0, 06 for both), and positively with S-ferritin, TSAT and CRP levels; associations of which all have been demonstred previously in a context of CKD [9, 19, 25]. The baseline H/F ratio, which was higher in the VD group, possibly due to a high proportion of women in whom hepcidin levels are reported to be higher than in men for the specific age category, was not associated with either calcitriol or S-25 (OH) D levels (www.hepcidinanalysis.com).

The strength of this study is that it was carried out in a double-blind, controlled fashion with systematic data collection enabling the assessment of nutritional VD to modify levels of HEP-25. In addition, S-HEP-25 was analyzed by mass spectrometry, an accurate method that allows measurement of HEP-25 specifically rather than measurement of the total of all hepcidin isoforms [7, 84]. Nevertheless, there are some limitations to the present study. This was a post-hoc analysis based on a randomized, controlled study in which data was collected with the primary aim of assessing the modifying effect of nutritional VD on PTH levels and the protocol was not adapted to assess an effect of VD on HEP-25 and anemia. Further, no power calculation was performed which may have limited its statistical power.

In conclusion, daily supplementation for 12 weeks with a high, daily dose of native VD did not entail any discernible effects on markers of hepcidin, erythropoiesis, or iron status when applying a cut-off of 75 nmol /L. However, observations based on subgroup analyses showed VD supplementation to associate with beneficial effects on erythropoiesis and iron availability in CKD patients with low baseline 25(OH)D levels. Whether VD could play a role as adjunctive treatment in patients with renal anemia in carefully selected patients can however only be answered by prospectively, PBO-controlled studies. In contrast to the findings of the low 25(OH)D group, a high-dose VD regimen associated with a decrease in hepcidin levels in the patients with elevated 25(OH)D levels at baseline, possibly a manifestation of an unfavourable effect on iron availability.

## Data Availability

Data base can be made available upon reasonable request to the corresponding author.
